# Science in Vanderbilt University

**Published:** 1878-08

**Authors:** 


### Science in Vanderbilt University.

It will be seen that Prof. Winchell has simply accepted the views that now prevail in the scientific world regarding the time that men have inhabited the earth. The old notions upon the subject are now no longer entertained by intelligent men, because the scientific evidence is overwhelmingly against them. How long the human race has occupied the globe is an open question, but two things are settled: 1. That man has a much higher antiquity than theology has been in the habit of teaching, and has been currently believed; and, 2. That the investigation belongs entirely to science, and must be pursued and determined on the basis of scientific evidence. Prof. Winchell argues the subject strictly as a scientist, but in no spirit of antagonism to religion or to the authority of the Bible. His argument, indeed, is rather an attempt to harmonize the teachings of Scripture with the conclusions of science; a task which he had previously undertaken in a more neral way, by the publication of an able book on "The Reconciliation of Science and Religion." But he has met with the not unusual treatment of peace-makers, who miscalculate the temper of the strife they would compose. The stupid Southern Methodists that control the university, it seems, can learn nothing. The fight over the antiquity of the earth has but just closed. The theologians battled long and fiercely against the geologists on this question, but have been so utterly routed that hsrdly a man of them can now be found who holds to the old belief. Prof. Silliman led the scientific movement upon that subject in this country, and so profound was the theological alarm that eminent doctors of divinity implored him with tears to desist from the impious crusade, because, if successful, it would be certain to destroy the Christian college with which he was associated. That institution had the good sense not to disturb the distinguished teacher in his work, but to abide by the results of investigation; and who can now be found so foolish as to regret its course? But half a century later, when an analogous question arises, Vanderbilt University adopts a different policy, follows the exploded precedents of past centuries, and puts forth its power to muzzle, repress, silence, and discredit the independent teachers of scientific truth.—PROF. YOUMANS, *in Popular Science Monthly for August.*

